# Correlation of *Moraxella catarrhalis* macrolide susceptibility with the ability to adhere and invade human respiratory epithelial cells

**DOI:** 10.1080/22221751.2022.2108341

**Published:** 2022-08-31

**Authors:** Ya-li Liu, Rui Ding, Xin-miao Jia, Jing-jing Huang, Shuying Yu, Hiu Tat Chan, Wei Li, Lei-li Mao, Li Zhang, Xin-yao Zhang, Wei Wu, An-ping Ni, Ying-chun Xu

**Affiliations:** a Department of Laboratory Medicine, State Key Laboratory of Complex Severe and Rare Diseases, Peking Union Medical College Hospital, Chinese Academy of Medical Science and Peking Union Medical College, Beijing, People’s Republic of China; b Graduate School, Chinese Academy of Medical Sciences and Peking Union Medical College, Beijing, People’s Republic of China; c Beijing Key Laboratory for Mechanisms Research and Precision Diagnosis of Invasive Fungal Diseases, Beijing, People’s Republic of China; d Medical Research Center, State Key laboratory of Complex Severe and Rare Diseases, Peking Union Medical College Hospital, Chinese Academy of Medical Sciences and Peking Union Medical College, Beijing, People’s Republic of China; e Department of Physiology, Anatomy and Microbiology, La Trobe University, Bundoora, Australia

**Keywords:** *Moraxella catarrhalis;* virulence genes, adhesion, invasion, cytokines, pathogenicity, macrolide

## Abstract

Recently, the prevalence of macrolide-resistant *Moraxella catarrhalis* has been reported, especially among Chinese children. The fitness cost of resistance is reported to render the resistant bacteria less virulent. To investigate the correlation between macrolide susceptibility of *M. catarrhalis* and pathogenicity, the whole genome of 70 *M. catarrhalis* isolates belonging to four clonal complexes with different macrolide susceptibilities was sequenced. The gene products were annotated with the Gene Ontology terms. Based on 46 extracted essential virulence genes, 19 representative isolates were selected to infect type II alveolar cells (A549 cells). The ability of these isolates to adhere and invade human epithelial cells and to produce cytokines was comparatively analysed. Furthermore, mice were infected with a pair of *M. catarrhalis* isolates with different pathogenic behaviours and macrolide susceptibilities to examine pulmonary clearance, histological findings, and the production of cytokines. The percentages of annotations for binding, metabolic process, cellular process, and cell were non-significantly different between the macrolide-resistant and macrolide-susceptible groups. The presence of *uspA2*, *uspA2H*, *pilO*, *lbpB*, *lex1*, *modM*, *mboIA*, and *mboIB* significantly differed among the four clonal complexes and macrolide susceptibility groups. Furthermore, compared with those in macrolide-susceptible isolates, the adhesion ability was stronger (*P* = 0.0019) and the invasion ability was weaker (*P* < 0.0001) in the macrolide-resistant isolates. Mouse experiments revealed that pulmonary macrophages elicit immune responses against *M. catarrhalis* infection by significantly upregulating the Csf2, Il4, Il13, Il1b, Il6, Tnf, and Il18. Therefore, *M. catarrhalis* populations exhibited diverse pathogenicity *in vitro* and *in vivo*.

## Introduction

*Moraxella catarrhalis* is a major etiological agent for acute otitis media and acute exacerbation of chronic obstructive pulmonary disease. Additionally, *M. catarrhalis* can cause other serious infections, such as meningoencephalitis [1] and community-acquired pneumonia [2]. Macrolides are commonly used to treat respiratory illnesses*,* including those caused by *M. catarrhalis* [3]. Recent studies have reported the prevalence of macrolide-resistant *M. catarrhalis* [4–6]. The fitness cost of antibiotic resistance is a phenomenon in which antibiotic-resistant mutants exhibit decreased fitness and virulence without an apparent reason [7,8]. Previously, we had demonstrated that macrolide-resistant isolates were highly concentrated in the CC449 (CCN10) and CC363 clonal complexes [9,10], while macrolide-susceptible isolates were highly concentrated in the CC224 and CC446 (CCN08) clonal complexes [9]. We hypothesized that populations of *M. catarrhalis* isolates with different macrolide susceptibilities exhibit differential pathogenic behaviours, such as the ability to adhere and invade human epithelial cells.

Some virulence factors, including UspA1, UspA2, UspA2H, OMPCD, OMP *M. catarrhalis* adherence protein (McaP), and *Moraxella* lipooligosaccharide (LOS), have been previously reported [11–14]. These virulence factors promote serum resistance to evade humoral immunity. Additionally, these virulence factors function as *M. catarrhalis* adhesins and bind to different human epithelial cell lines [15]. The interaction between *M. catarrhalis* and human-derived epithelial cell lines is multifactorial and involves the simultaneous activities of multiple adhesins [15]. We predict that various combinations of multiple adhesins co-ordinate and act differently in *M. catarrhalis* isolates with different backgrounds (such as the macrolide-resistant and macrolide-susceptible groups), conferring differential abilities to adhere and invade human epithelial cells.

To confirm this hypothesis, 70 *M. catarrhalis* isolates with different macrolide susceptibilities, including those belonging to CC449, CC363, CC446, and CC224, were sequenced. The gene products were annotated with Gene Ontology (GO) terms. Based on the 46 extracted essential virulence genes, 19 isolates with 17 unique combinations of virulence genes were selected to infect type II alveolar cells (A549 cells). The abilities of the isolates to adhere and invade human epithelial cells and produce cytokines were comparatively evaluated. Based on the results of this experiment, mice were infected with a pair of *M. catarrhalis* isolates with different pathogenic behaviours and macrolide susceptibilities to analyse pulmonary clearance, histological changes, and the production of cytokines. Macrolide-resistant and macrolide-susceptible *M. catarrhalis* isolates exhibited differential abilities to adhere and invade human epithelial cells with several important virulence genes facilitating this process. Additionally, mouse experiments suggested that pulmonary macrophages mediate the rapid immune response to *M. catarrhalis* infection. This immune response involved the upregulation of cytokines, such as Csf2, Il4, Il13, Il1b, Il6, Tnf, and Il18.

## Materials and methods

### Bacterial strains

Seventy non-duplicate clinical *M. catarrhalis* isolates were collected from the sputum or broncho-alveolar lavage of adult patients (>18 years of age) diagnosed with lower respiratory tract infection and the sputum or ear purulent secretion of children diagnosed with lower or upper respiratory tract infections between 2010 and 2017. Of these, 22, 40, and 8 isolates were obtained from the Peking Union Medical College Hospital (PUMCH), the First Affiliated Hospital of Xiamen University, and other hospitals in China, respectively. The isolates were identified using the Bruker Biotyper matrix-assisted laser desorption ionisation-time of flight mass spectrometry system (Biotyper version 3.1 software, Bruker Daltonics, Billerica, Germany). The susceptibilities of 70 isolates to erythromycin and azithromycin were examined using the Kirby-Bauer method and E-test. Previously, we had performed 23S rRNA gene and multilocus sequence typing analyses [9]. In this study, 70 isolates belonged to the following four clonal complexes: CC449 (n = 14; 14 macrolide-resistant isolates with A2330T mutation), CC363 (n = 18; 13 macrolide-resistant isolates with A2330T mutation and 5 macrolide-susceptible isolates without A2330T mutation), CC446 (n = 13; 13 macrolide-susceptible isolates without A2330T mutation), and CC224 (n = 25; 3 macrolide-resistant isolates with A2330T mutation, 22 macrolide-susceptible isolates without A2330T mutation). The isolates were stored at −80°C.

### Genomic DNA extraction, sequencing, assembly, and annotation

Genomic DNA was extracted using the TIANamp bacteria genomic DNA kit (TiangenBiotechCo. Ltd., Beijing, China) and subjected to shotgun sequencing using Illumina Genome Analyzer IIx technology. Paired-end libraries were prepared from 5 μg of isolated genomic DNA using the TruSeq DNA sample prep kit A (Illumina Inc., San Diego), following the manufacturer's instructions. Genomic paired-end libraries were sequenced with a read length of 2 × 150 nucleotides using an Illumina GAIIx instrument, following the manufacturer's instructions. Image analysis and base calling were performed using the standard Illumina pipeline. The raw Illumina sequencing reads were trimmed at a threshold of 0.01 (Phred score of 20) using Fastx-toolkit (http://hannonlab.cshl.edu/fastx_toolkit/commandline.html). SPAdes [16] was used for assembling the filtered reads with parameters “-k 33,45,55 –careful –cov-cutoff 5”. All assembled contig sequences were annotated with Prokka (Tools used in Prokka: Prodigal for protein-coding features (CDS), RNAmmer for rRNA, Aragorn for tRNA) [17].

### Virulence gene analysis

The sequences of 46 virulence genes, which were retrieved from literature, were downloaded from the Virulence Factor Database (VFDB, http://www.mgc.ac.cn/cgi-bin/VFs/v5/main.cgi, VFDB_setB_nt.fas.gz) and National Centre for Biotechnology Institute (NCBI). The detailed gene information is shown in Table S1. The virulence genes in the sequenced strains were predicted using SRST2 [18] and confirmed using Basic Local Alignment Search Tool (BLAST) with the following parameters: identity > 50% and coverage > 50%.

### Gene homology analysis and functional enrichment

Gene homology analysis was performed based on the pan-genome results using Roary with the parameters “-e create a multiFASTA alignment of core genes using PRANK; -n fast core gene alignment with MAFFT” [19]. The gene distribution file was obtained from the “gene_presence_absence.csv” and “gene_presence_absence.Rtab” files. Specific genes of a certain group were obtained using the χ^2^-test (*P*-value < 0.05) for categorical data transformed from the gene distribution obtained from Roary. The gene functions were further annotated using Blast2GO to obtain the enriched GO terms (https://www.blast2go.com/). GO annotation results were visualized and compared using WEGO [20,21]. Significant (*P* < 0.05) GO terms were determined using the Pearson χ^2^ test.

### Cell lines

A549 epithelial cells (type II alveolar cells) were obtained from American Type Culture Collection and cultured in Ham's F12 medium (Gibco) supplemented with 10% (v/v) heat-inactivated fetal calf serum (Gibco), 100 U/mL penicillin, and 0.1 mg/mL streptomycin (Solaribo, China). Cells were cultured until 95%–100% confluency in 25-cm^2^ flasks, trypsinised, and transferred to 24-well plates (Thermo Fisher Scientific™, Waltham, MA, USA) to perform the adhesion and invasion assays.

### Adhesion assay

To examine the effect of virulence genes on the ability of *M. catarrhalis* isolates to adhere and invade human respiratory epithelial cells, 19 isolates with 17 unique combinations of virulence genes were selected from the 70 isolates used in this study.

To determine the optimal ratio of bacterium to host cells for application in the adhesion and invasion assays, the following five multiplicity of infection (MOI) ratios were examined: 2:1, 4:1, 10:1, 20:1, and 200:1. The test MOI ratios did not significantly affect bacterial adhesion to A549 cells but markedly affected the invasion of *M. catarrhalis* isolates into A549 cells (data not shown). The optimal MOI with detectable bacterial numbers and low variability was 10. Therefore, an MOI of 10 was used in the subsequent adhesion and invasion assays.

The adhesion assay was performed as previously described [22] with some modifications. Briefly, A549 cells were plated in 24-well cell culture plates (1 × 10^5^ cells/well) and incubated with an appropriate MOI (10) of *M. catarrhalis* for 4 h. Non-adherent bacteria were removed by washing with phosphate-buffered saline (PBS, PH 7.2-7.4). Cells were lysed with 200 µL of 0.25% trypsin (Sigma) at 37°C for 10 min and 300 µL of 0.1% saponin (Sigma) for 10 min. The lysates were transferred to 1.5 mL Eppendorf tubes and mixed vigorously for 30 s. Aliquots of the samples were serially diluted (1 × 10^−3^, 1 × 10^−4^, and 1 × 10^−5^) and plated on blood agar plates (100 µL/plate) containing 5% sheep blood to determine the number of adherent bacteria. All assays were performed in six replicates. At least three independent experiments were performed.

### Invasion assay

The susceptibility of 70 *M. catarrhalis* isolates to gentamycin was examined. All isolates were susceptible to gentamycin. The results of the *in vitro* time-kill assay revealed that gentamycin exhibited bactericidal activity (99.9% killing) at a concentration of 300 µg/mL (treatment duration: 1 h). Therefore, 300 µg/mL gentamycin was used in the subsequent experiments in this study.

From the 70 isolates used in this study, 19 isolates with 17 unique combinations of virulence genes were selected to examine their abilities to invade A549 cells. The experimental procedure of the invasion assay was similar to that of the adhesion assay [22], except 300 µg/mL gentamycin was added to the infected monolayer cells before lysing with 0.25% trypsin and 0.1% saponin to kill any remaining adherent extracellular bacteria. The cells were infected with *M. catarrhalis* at density of 2 × 10^5^ (MOI = 2), 4 × 10^5^ (MOI = 4), 1 × 10^6^ (MOI = 10), 2 × 10^6^ (MOI = 20), or 2×10^7^ CFU/well (MOI = 200) for 4 h. All assays were performed in five or six parallel wells. At least three independent experiments were performed. Results of representative experiments are presented as mean (colony-forming unit (CFU) per well) ± standard deviation.

### Electron microscopy

A549 cells were plated in 24-well cell culture plates (1 × 10^5^ cells/well) and incubated with *M. catarrhalis* [73-OR (CC446) or 35-OR (CC363)] at MOI 10 for 4 h. Non-adherent bacteria were removed by washing with PBS. Cells were lysed with 200 µL of 0.25% trypsin (Sigma) at 37°C for 10 min. The lysate was centrifuged and washed twice with PBS. The infected A549 cells were fixed in 2.5% glutaraldehyde in phosphate buffer (0.06 M) for up to 2 days (pH 7.3). Cells were prepared for electron microscopy as previously described [22]. The distribution of *M. catarrhalis* isolates outside and inside A549 cells after infection for 4 h was determined at a magnification of ×3000. Meanwhile, the lamellipodia enclosing *M. catarrhalis* isolates after 4 h of infection and different phases of macropinocytotic ingestion were observed at a magnification of ×15000.

### Pulmonary clearance model

The mouse pulmonary clearance model was established as described previously [23–26]. The animal study was approved by the Institute of Microbiology, Chinese Academy of Sciences Animal Care and Use Committee (project number: APIMCAS2021144). C57/BL6J male mice (aged 8–10 weeks) were obtained from Jackson Laboratories (Bar Harbour, ME) and housed under standard pathogen-free conditions in an animal facility at the Institute of Microbiology, Chinese Academy of Sciences. Mice (n = 5–7 per group) were intraperitoneally anesthetised using isoflurane. After the trachea was surgically exposed, the mice in the control (PBS-treated), 73-OR (macrolide-susceptible isolate)-infected, and 35-OR (macrolide-resistant isolate)-infected groups were intratracheally injected with PBS, 10^8^ CFU of 73-OR *M. catarrhalis*, and 10^8^ of 35-OR *M. catarrhalis*, respectively. The bacterial suspension was prepared in 50 µL PBS. The surgical wound was closed using 5.0 silk sutures, and the animals were allowed to recover. At 3 h post-challenge, the animals were sacrificed via CO_2_ exposure. The blood and lung tissues were collected. The number of white blood cells (WBCs) in the blood was measured using an automated haematological analyser Sysmex Xs-800i. The lungs were homogenized on ice in 1 mL of PBS (PH 7.2-7.4) using a tissue homogenizer. Next, the samples were serially diluted and plated on blood agar plates (100 µL/plate) containing 5% sheep blood (two replicates). After incubation overnight at 37°C and 5% CO_2_, the CFU was determined via manual counting.

### Histological analysis

Mice (n = 5–7/group) were challenged with PBS or 10^8^ CFU of *M. catarrhalis* (73-OR or 35-OR) in 50 µL PBS as described above. To examine pulmonary inflammation, both lungs were inflated with 10% buffered formaldehyde and perfuse-fixed as previously described [23]. The fixed lungs were embedded in paraffin, stained with haematoxylin and eosin, and examined under a microscope. The lung injuries, including alveolar structure damage and inflammatory cell infiltration, were scored in a blinded manner. Histological changes in the lung tissue sections of different groups were compared at magnifications of ×100 and ×400.

### Cytokine profile analysis

Lung tissues and plasma of mice were collected after challenge with PBS or 10^8^ CFU of *M. catarrhalis* (73-OR or 35-OR). The levels of Ifng, Il12p70, Il13, Il1b, Il2, Il4, Il5, Il6, Il18, Il10, Il17a, Il22, Il23, Il27, Il9, Tnf, and Csf2 were measured using the Th1/Th2/Th9/Th17/Th22/Treg Cytokine 17-Plex Mouse ProcartaPlex™ Panel (Invitrogen, Thermo Fisher Scientific, USA).

### Statistical analysis

GO annotation results were visualized and compared using WEGO [20,21]. Significant (*P* < 0.05) GO terms were determined using Pearson's χ^2^-test (or Fisher's exact test, when appropriate). The prevalence of virulence genes among the four clonal complexes and the two macrolide susceptibility groups was compared using Pearson's χ^2^-test (or Fisher's exact test, when appropriate) for categorical data (Table 1). The levels of cytokines and bacterial growth between the macrolide-susceptible and macrolide-resistant groups were compared using the two-tailed Mann–Whitney test, while those between the four clonal complexes were compared using one-way analysis of variance (ANOVA). Pulmonary clearance and WBC counts in mice belonging to the control, 73-OR-infected, and 35-OR-infected groups were compared using one-way ANOVA. Differences were considered significant at *P* < 0.05.

**Table 1.** Prevalence of 13 virulence genes in four clonal complexes and two macrolide susceptibility Moraxella catarrhalis groups.

Genes	Prevalence of genes in four clonal complexes				*P*-value	Prevalence of genes in two macrolide susceptibility groups		*P*-value
	CC224 (n = 25)	CC449 (n = 14)	CC446 (n = 13)	CC363 (n = 18)		Macrolide-resistant Group (n = 30)	Macrolide-susceptible Group (n = 40)	
*uspA1*	22; 88.0%	13; 92.9%	9; 69.2%	17; 94.4%	0.1715	28; 93.3%	33; 82.5%	0.2828
*uspA2*	13; 52.0%	13; 92.9%	1; 7.7%	15; 83.3%	< 0.0001	24; 80.0%	18; 45.0%	0.0036
*uspA2H*	15; 60.0%	12; 85.7%	2; 15.4%	13; 72.2%	0.0013	21; 70.0%	21; 52.5%	0.2175
*MID_Hag*	25; 100%	14; 100%	12; 92.3%	18; 100%	0.217	30; 100%	39; 97.5%	1.0000
*pilD*	25; 100%	14; 100%	12; 92.3%	18; 100%	0.217	30; 100%	39; 97.5%	1.0000
*pilA*	23; 92.0%	14; 100%	12; 92.3%	18; 100%	0.4503	27; 90%	40; 100%	0.0742
*pilO*	25; 100%	14; 100%	10; 76.9%	18; 100%	0.0033	30; 100%	37; 92.5%	0.2547
*lbpB*	2; 8.0%	14; 100%	13; 100%	18; 100%	< 0.0001	27; 90%	20; 50%	0.0006
*tbpB*	22; 88.0%	14; 100%	13; 100%	18; 100%	0.1304	29; 96.7%	38; 95.0%	1.0000
*lex1*	3; 12.0%	14; 100%	13; 100%	18; 100%	< 0.0001	27; 90%	21; 52.5%	0.0008
*modM*	0; 0.0%	0; 0.0%	13; 100%	18; 100%	< 0.0001	13; 43.3%	18; 45.0%	1.0000
*mboIA*	25; 100%	0; 0.0%	13; 100%	18; 100%	< 0.0001	16; 53.3%	40; 100%	< 0.0001
*mboIB*	25; 100%	0; 0.0%	13; 100%	18; 100%	< 0.0001	16; 53.3%	40; 100%	< 0.0001

### Ethical statement

Only de-identified clinically obtained bacterial isolates were used in this study. No human subjects participated in this study. The study was approved by the Human Research Ethics Committee of PUMCH (No. JS-2304).

## Results

### Comparative analysis of functional annotations

**Figure 1.**
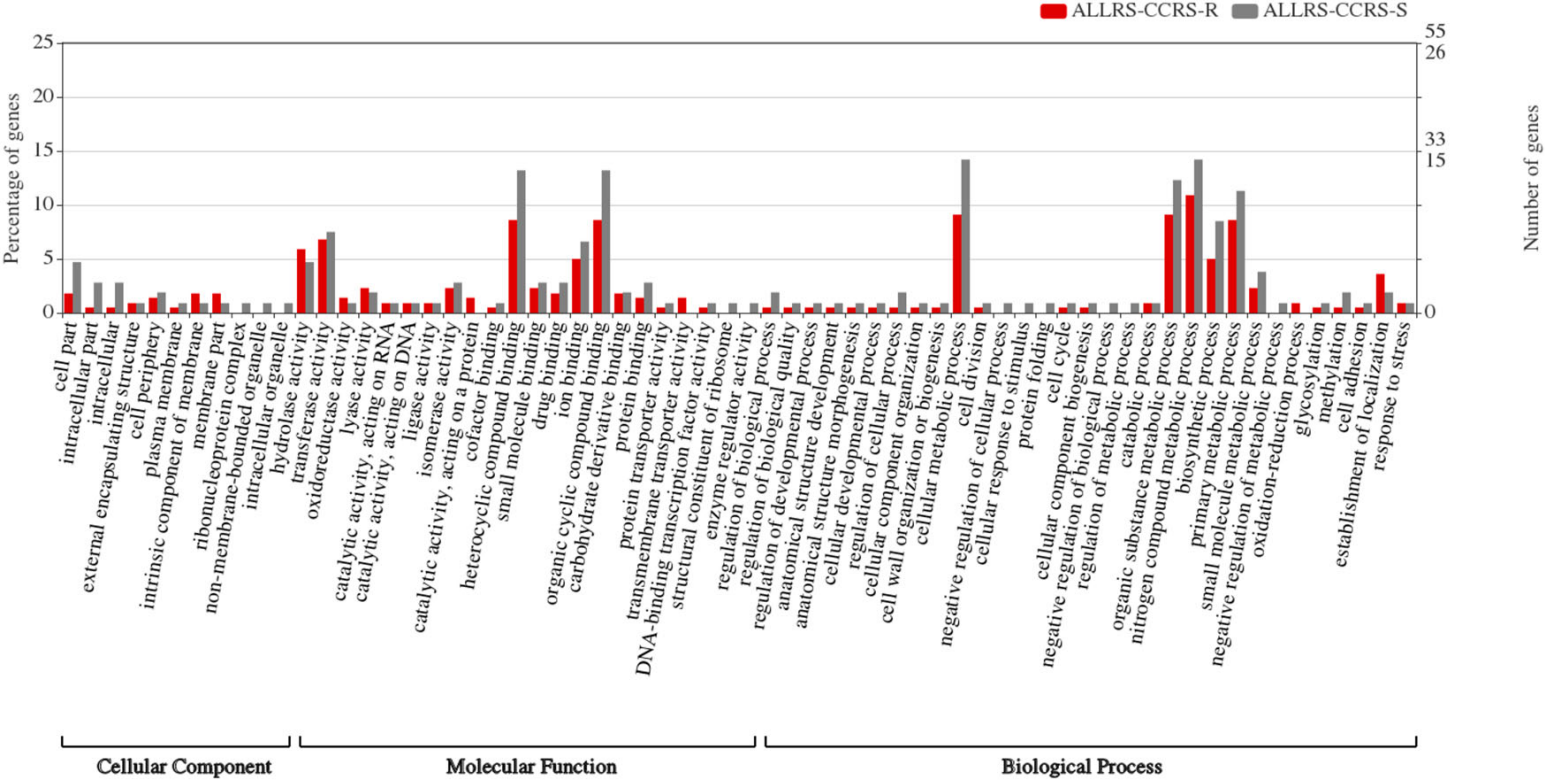
Comparison of functional annotations in the macrolide-resistant and macrolide-susceptible *Moraxella catarrhalis* groups.

In this study, 220 and 106 genes were extracted from the macrolide-resistant (n = 30) and macrolide-susceptible groups (n = 40), respectively, as they exhibited significant and differential prevalence between the two groups. Next, 91 of the 220 genes and 57 of the 106 genes were annotated to GO terms (Figure 1). The GO term with the highest number of assigned uni-genes in the molecular function (MF) category was catalytic activity, followed by binding, transporter activity, transcription regulator activity, structural molecule activity, and molecular function regulator. In the biological process (BP) category, the term with the most uni-genes was metabolic process, followed by cellular process, localization, response to stimulus, biological regulation, regulation of biological process, developmental process, cellular component organization or bio-genesis, biological adhesion, and negative regulation of biological process. Meanwhile, in the cellular component (CC) category, the term with the most uni-genes was cell, followed by membrane, membrane part, protein-containing complex, and organelle.

The following annotations were non-significantly different between the macrolide-resistant and macrolide-susceptible groups: MF category, binding (macrolide-resistant group (28 (12.7%)) vs. macrolide-susceptible group (21 (19.8%)); *P *= 0.13); BP category, metabolic process (macrolide-resistant group (28 (12.7%)) vs. macrolide-susceptible group (18 (17.0%)); *P *= 0.38) and cellular process (macrolide-resistant group (20 (9.1%)) vs. macrolide-susceptible group (16 (15.1%)); *P *= 0.15); CC category, cell (macrolide-resistant group (5 (2.3%)) vs. macrolide-susceptible group (6 (5.7%)); *P *= 0.20) (Figure 1). Cell in the CC category was characterized by increased percentages of annotations in the macrolide-susceptible group.

### Comparative analysis of virulence gene prevalence

**Figure 2.**
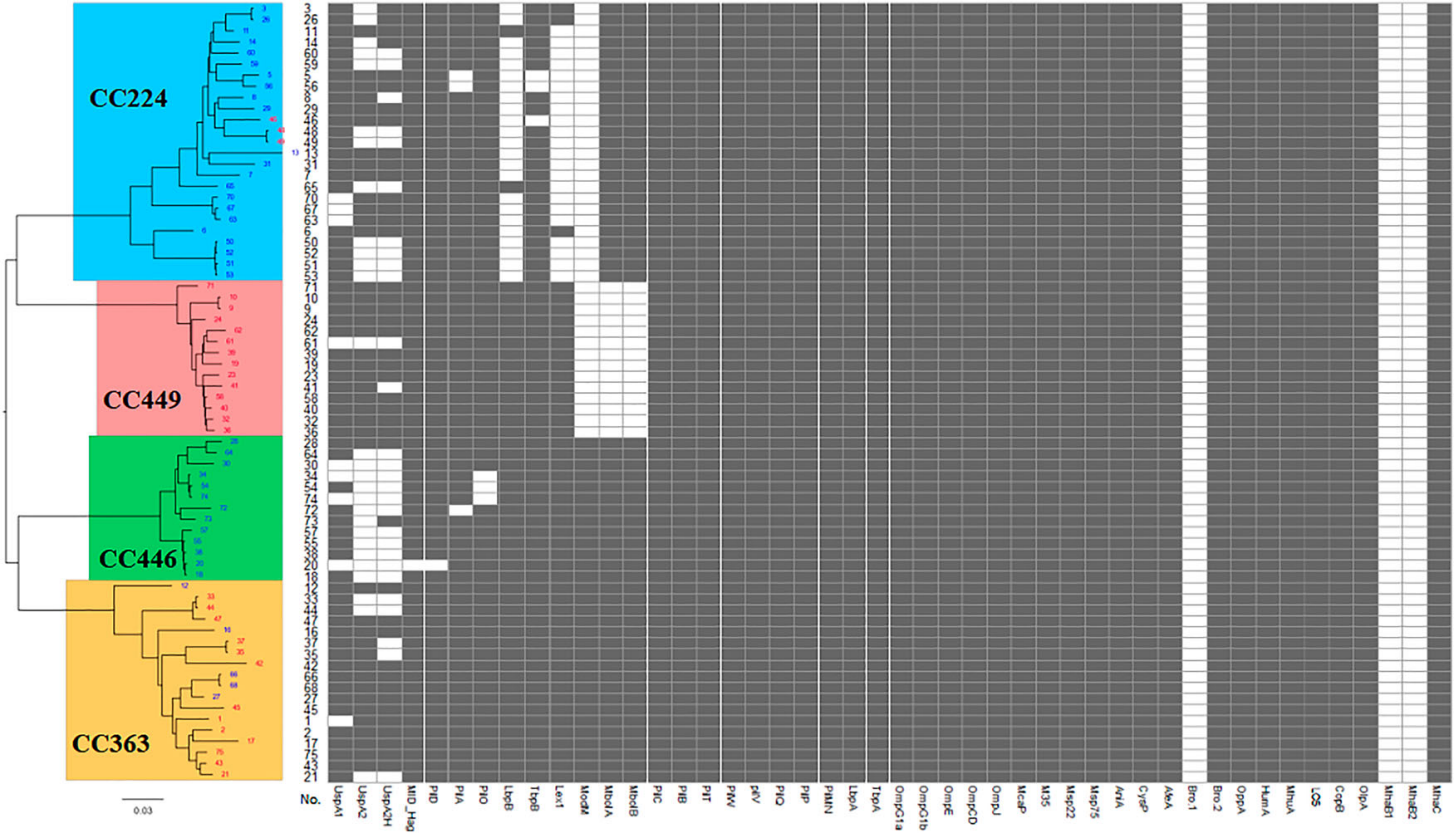
Comparison of virulence gene prevalence between four clonal complexes and between macrolide-resistant and macrolide-susceptible *Moraxella catarrhalis* groups. Macrolide-resistant and macrolide-susceptible *M. catarrhalis* isolates are shown in red and blue fonts, respectively. Grey and blank bars represent the presence and absence of genes, respectively.

Forty-six virulence gene sequences mentioned in previous studies [15,27,28] were downloaded from VFDB and NCBI. The prevalence of these virulence genes in the 70 sequenced isolates was predicted using SRST217 and confirmed using BLAST (identity > 50% and coverage > 50%). As shown in Figure 2, the distribution of 33 virulence genes (*pilC*, *pilB*, *pilT*, *pilW*, *pilV*, *pilQ*, *pilP*, *pilMN*, *lbpA*, *tbpA*, *ompG1a*, *ompG1b*, *ompE*, *ompCD*, *ompJ*, *mcaP*, *m35*, *msp22*, *msp75*, *aniA*, *cysP*, *afeA*, *bro1*, *bro2*, *oppA*, *humA*, *mhuA*, *los*, *copB*, *olpA*, *mhaB1*, *mhaB2*, and *mhaC*) was identical among the 70 isolates. However, the prevalence of 13 virulence genes varied between the four clonal complexes, as well as between the macrolide-resistant and macrolide-susceptible *M. catarrhalis* groups. The specific distribution of various genes is as follows (Table 1): *mboIA* and *mboIB*, specifically and completely absent in CC449 (*P* < 0.0001); *modM*, specifically and completely absent in CC224 and CC449 (*P* < 0.0001); *lbpB* and *lex1*, specifically and mostly absent in CC224 (*P* < 0.0001). Furthermore, *pilO* was absent in three isolates of CC446 and the difference was significant (*P* = 0.0033). *uspA2* and *uspA2H* were absent in all four clonal complexes. The highest number of isolates lacking *uspA2* and *uspA2H* was observed in CC446, followed by CC224, CC363, and CC449 (*P* < 0.05) (Table 1). Meanwhile, the prevalence of *uspA2*, *lbpB*, *lex1*, *mboIA*, and *mboIB* was significantly different between the macrolide-resistant and macrolide-susceptible *M. catarrhalis* groups (Table 1). *uspA2, lbpB*, and *lex1* were mainly absent in the macrolide-susceptible group, whereas *mboIA* and *mboIB* were absent only in the macrolide-resistant group.

### Comparative analysis of adhesion and invasion

**Figure 3.**
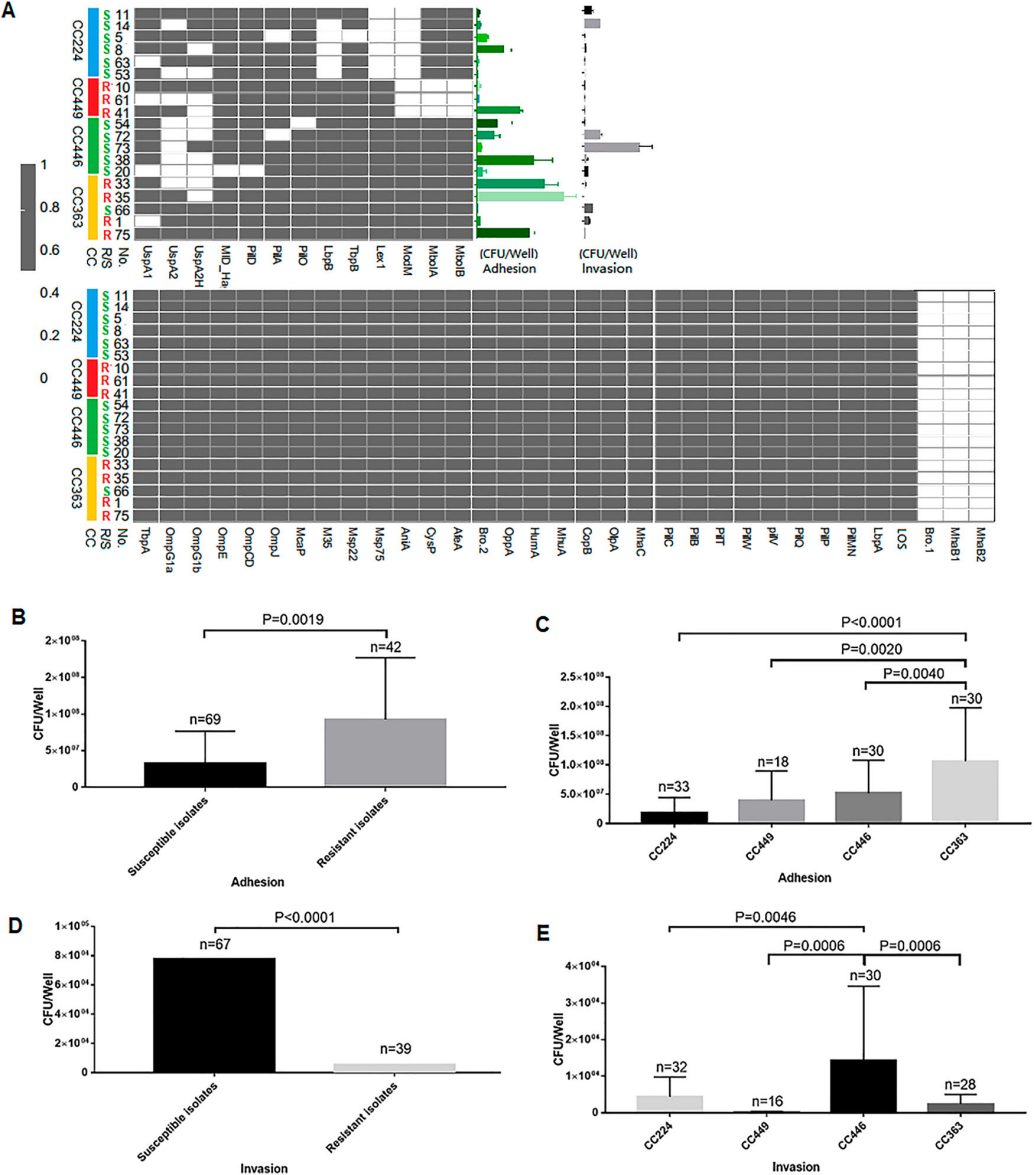
Comparison of adhesion and invasion abilities of 19 *Moraxella catarrhalis* isolates with different virulence gene combinations. R, macrolide-resistant; S, macrolide-susceptible. (A) The distribution of different virulence genes between four clonal complexes and between macrolide-resistant and macrolide-susceptible *M. catarrhalis* groups and its relevance to bacterial adhesion and invasion. Grey and blank bars represent the presence and absence of genes, respectively. (B and D) Comparison of *M. catarrhalis* adhesion and invasion abilities between the macrolide-resistant and macrolide-susceptible groups and (C and E) between four clonal complexes. The levels of cytokines and bacterial growth between the macrolide-susceptible and macrolide-resistant groups were analysed using the two-tailed Mann-Whitney test, while those between the four clonal complexes were analysed using one-way analysis of variance. Differences were considered significant at *P* < 0.05.

The prevalence of the virulence genes varied depending on the clonal complex and macrolide susceptibility. Hence, 19 isolates with 17 unique combinations of virulence genes were selected from the four clonal complexes (CC224, n = 6; CC449, n = 3; CC446, n = 5; and CC363, n = 5). Compared with those in the macrolide-susceptible isolates, the adhesion ability was significantly stronger (*P *= 0.0019) and the invasion ability was significantly weaker (*P < *0.0001) in the macrolide-resistant isolates (Figure 3B and D). Additionally, the adhesion and invasion abilities were significantly different between the main clonal complexes. CC363, a major macrolide-resistant clonal complex, exhibited the highest host cell adhesion ability, followed by CC446 (a purely macrolide-susceptible clonal complex), CC449 (a purely macrolide-resistant clonal complex), and CC224 (a mostly macrolide-susceptible clonal complex) (Figure 3C). In contrast, CC446, a pure macrolide-susceptible clonal complex, exhibited the highest host cell invasion ability, followed by CC224 (a majorly macrolide-susceptible clonal complex), CC363 (a majorly macrolide-resistant clonal complex), and CC449 (a purely macrolide-resistant clonal complex) (Figure 3E).

**Figure 4.**
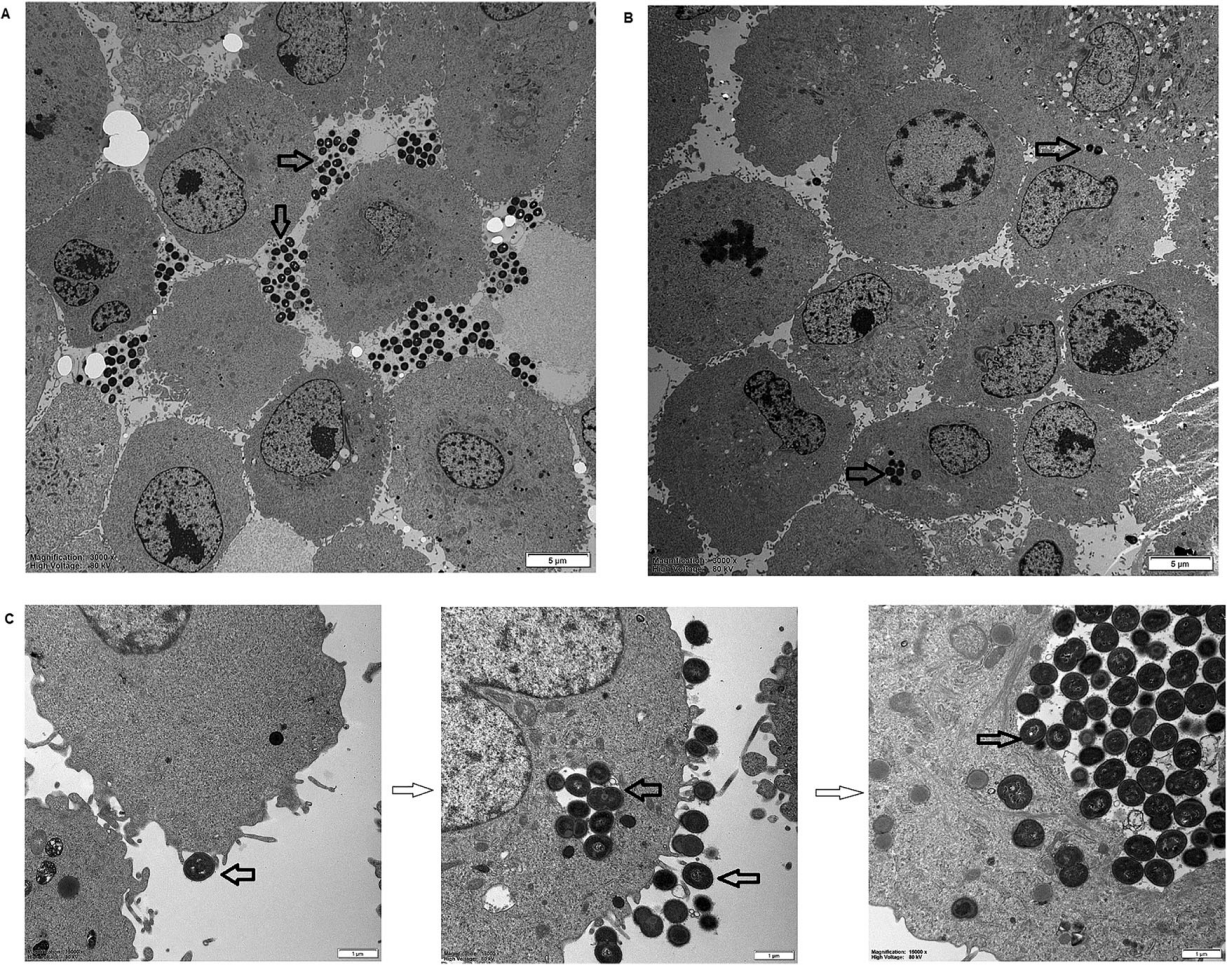
Transmission electron microscopy analysis of the ability of *Moraxella catarrhalis* to adhere and invade A549 cells. The distribution of *M. catarrhalis* 35-OR (resistant isolate) (A) and *M. catarrhalis* 73-OR (susceptible isolate) (B) outside and inside A549 cells at 4 h post-infection (magnification: ×3000). (C) Lamellipodia enclosing *M. catarrhalis* 73-OR (susceptible isolate) at 4 h post-infection and different phases of macropinocytotic ingestion (magnification: ×15000). *M. catarrhalis* isolates are shown using arrows. Scale bars: 5 (A and B) or 1 µm (C).

To further demonstrate the differential behaviours of the *M. catarrhalis* populations, one macrolide-resistant isolate (35-OR) with strong host cell adhesion ability and weak host cell invasion ability (mean number of adhering cells = 2.6 × 10^7^ CFU/well; mean number of invading cells = 5 CFU/well; [(number of invading cells/number of adhering cells) × 100] = 0.000019%) and one macrolide-susceptible isolate (73-OR) with a weak host cell adhesion ability and a strong host cell invasion ability (mean number of adhering cells = 1.7 × 10^7^ CFU/well; mean number of invading cells = 8.6 × 10^2^ CFU/well; [(number of invading cells/number of adhering cells) × 100] = 0.005170%) were selected to infect A549 cells (*P *= 0.0286). The cells were fixed and subjected to transmission electron microscopy (TEM) (Figure 4). The adherent *M. catarrhalis* 35-OR cells formed multicellular grape-like aggregates on the A549 cell surface (Figure 4A) but did not invade the cells. In contrast, some *M. catarrhalis* 73-OR cells adhered to the surface of A549 cells, while a visible number of isolates infiltrated the cells (Figure 4B). These results are consistent with those of bacterial growth analysis. TEM analysis confirmed that the epithelial cell monolayer formed lamellipodia and filopodia, extending toward and enclosing individual adherent bacterial cells (Figure 4C). Different phases of macropinocytotic ingestion are shown in Figure 4C.

### Effects of M. catarrhalis populations on animal models

**Figure 5.**
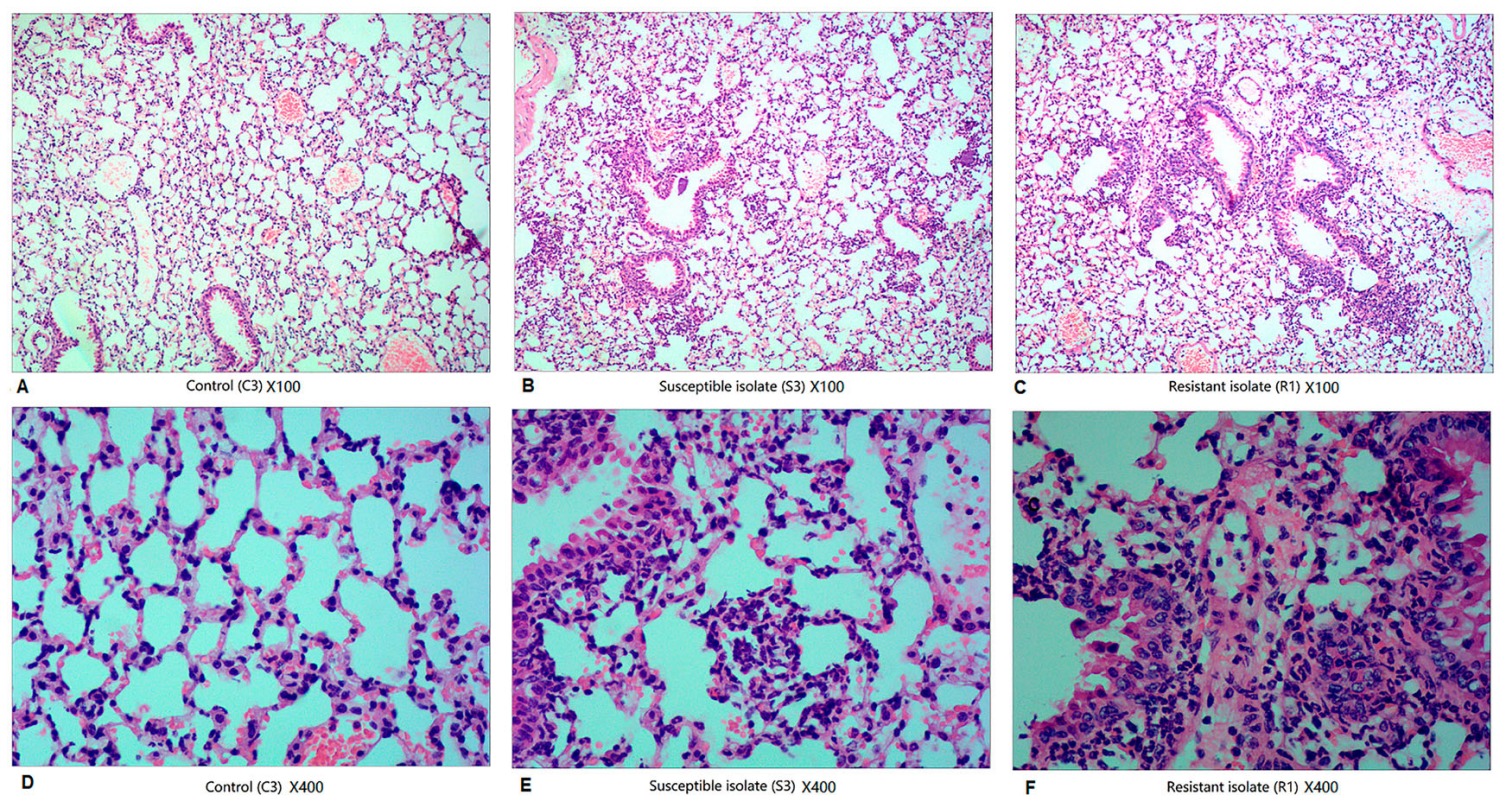
Histological changes in the haematoxylin and eosin-stained pulmonary tissue at 3 h post-infection (magnification: ×100 and ×400). Pulmonary inflammation in mice (n = 5–7 per group) challenged with phosphate-buffered saline (PBS), 10^8^ colony-forming units (CFU) of *Moraxella catarrhalis* 73-OR, and 10^8^ CFU of *M. catarrhalis* 35-OR. Representative lung tissue sections are shown. Pulmonary sections of control (C3) (magnification: ×100 (A) and ×400 (D)), mice (S3) challenged with 10^8^ CFU of *M. catarrhalis* 73-OR (susceptible isolate) (magnification: ×100 (B) and ×400 (E)), and mice (R1) challenged with 10^8^ CFU of *M. catarrhalis* 35-OR (resistant isolate) at 3 h post-infection (magnification: ×100 (C) and ×400 (F)).

Histological analysis (Figure 5) revealed that alveolar and bronchial structures were almost intact and visible in the three groups. Compared with that in the alveolar sputum of control mice (Figure 5A and D), the number of inflammatory cells, especially pulmonary macrophages and neutrophils, was higher in the alveolar sputum of 73-OR-infected (Figure 5B and E) or 35-OR-infected mice (Figure 5C and F). Immune cell infiltration and fibrous tissue hyperplasia were also observed. Moreover, inflammation severity in the 35-OR-infected group was higher than that in the 73-OR-infected group as evidenced by increased numbers of inflammatory cells and the manifestation of fibrous tissue hyperplasia.

**Figure 6.**
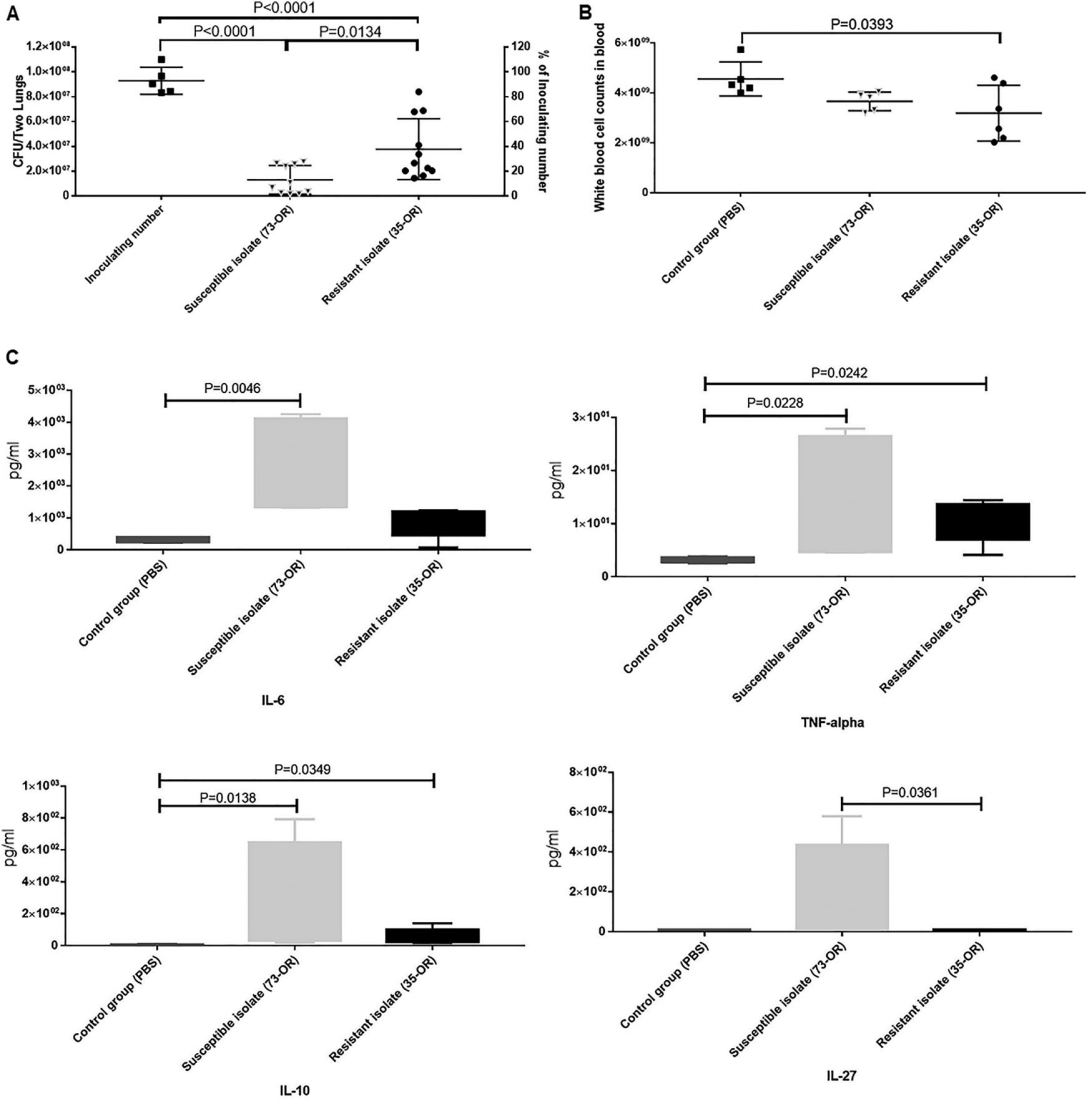
Bacterial counts in the lung tissue and white blood cell (WBC) counts and cytokine levels in the blood after *Moraxella catarrhalis* infection. Pulmonary clearance and systemic inflammatory changes in mice (n = 5–7 per group) challenged with phosphate-buffered saline (PBS), 10^8^ CFU of *M. catarrhalis* 73-OR, or 10^8^ CFU of *M. catarrhalis* 35-OR. (A) The bacterial counts in the 73-OR-infected and 35-OR-infected groups at 3 h post-infection were significantly (*P < *0.0001) lower than that at 0 h post-infection. The clearance of susceptible isolate (73-OR) was significantly rapid when compared with that of resistant isolate (35-OR) (*P *= 0.0134). (B) At 3 h post-infection, the WBC counts in the 73-OR-infected and 35-OR-infected groups were lower than those in the control group. In particular, the WBC counts significantly decreased in the 35-OR-infected group (*P = *0.0393). (C) At 3 h post-infection, the blood Il6, Il10, Il27, and Tnf levels in the 73-OR-infected and 35-OR-infected groups were significantly (*P < *0.05) higher than those in the control group, especially in the 73-OR-infected group. Pulmonary clearance and WBC counts in the control, macrolide-susceptible, and macrolide-resistant groups were compared using one-way analysis of variance. Differences were considered significant at *P* < 0.05.

Next, bacterial counts in the total lung tissue and WBC counts in the blood after *M. catarrhalis* infection were determined (Figure 6). The bacterial counts in the 73-OR-infected and 35-OR-infected groups at 3 h post-infection were significantly (*P < *0.0001) higher than those at 0 h post-infection (Figure 6A). The clearance of the 73-OR isolate was significantly rapid when compared with that of the 35-OR isolate (*P *= 0.0134). These results were consistent with the histological findings, which revealed severe inflammation in the 35-OR-infected group. Meanwhile, compared with those in the control group, the blood WBC counts were lower in the 73-OR-infected group and 35-OR-infected groups, especially in the 35-OR-infected group (*P = *0.0393) (Figure 6B).

### Cytokine profile analysis

**Figure 7.**
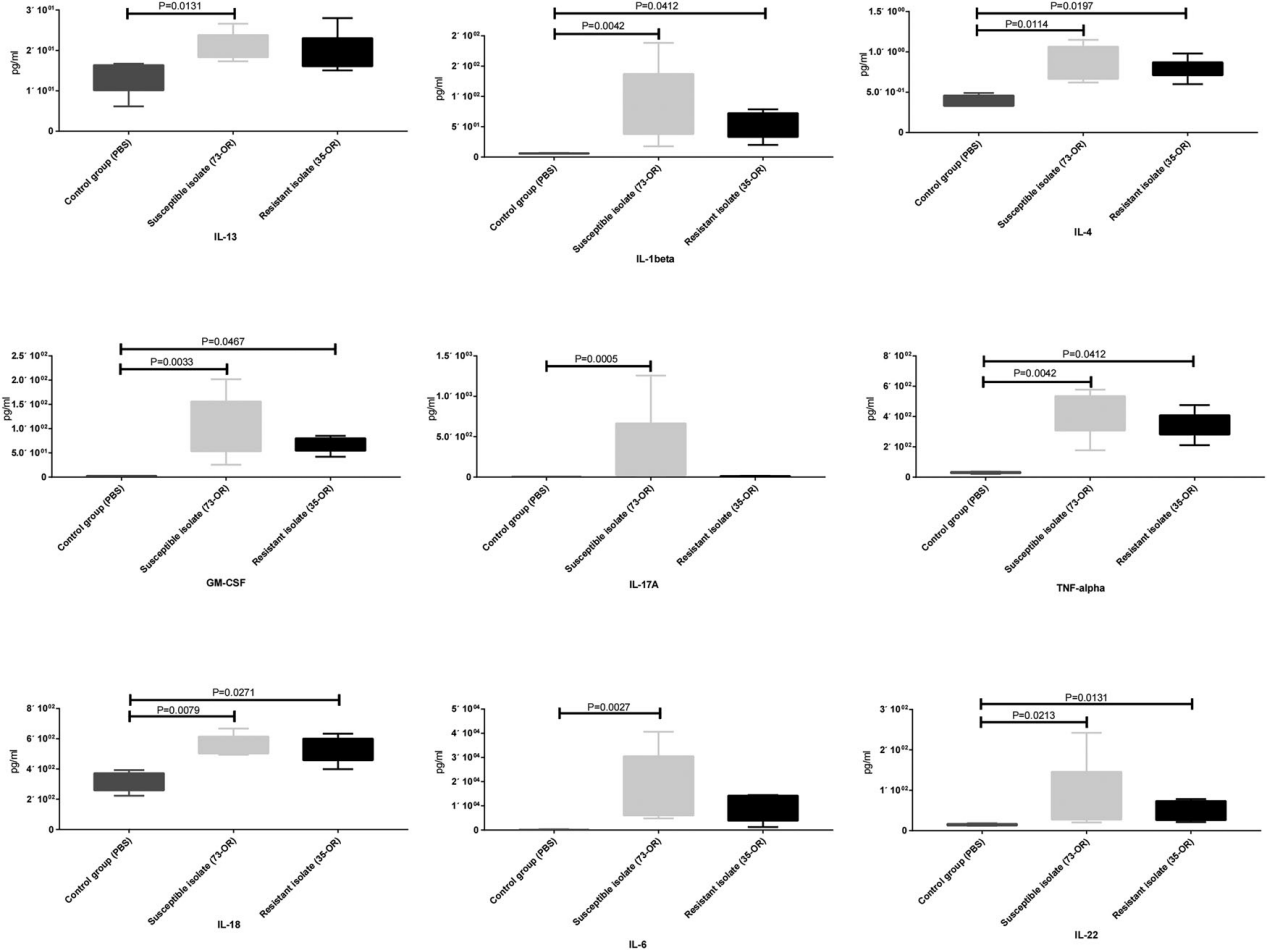
Pulmonary levels of cytokines in *Moraxella catarrhalis*-infected mice. Pulmonary inflammation in mice (n = 5–7/group) challenged with phosphate-buffered saline (PBS), 10^8^ colony-forming units (CFU) of *M. catarrhalis* 73-OR, and 10^8^ CFU of *M. catarrhalis* 35-OR. At 3 h post-infection, the Il13, Il1b, Il4, Il6, Il17a, Il18, Il22, Tnf, and Csf2 levels in the 73-OR-infected and 35-OR-infected groups were significantly (*P < *0.05) higher than those in the control group, especially in the 73-OR-infected group.

The plasma concentrations of cytokines were examined (Figure 6C). The Il6, Il10, Il27, and Tnf levels in the infected groups were significantly (*P* < 0.05) upregulated when compared with those in the control group. However, the levels of the other 13 cytokines were not significantly different between the two groups. Additionally, the cytokine levels in the 73-OR-infected group were non-significantly higher than those in the 35-OR-infected group. The pulmonary levels of cytokines were also examined (Figure 7). Compared with those in the control group, the pulmonary Il13, Il1b, Il4, Il6, Il17a, Il18, Il22, Tnf, and Csf2 levels were significantly upregulated (*P* < 0.05) in the infected group. However, the pulmonary levels of eight cytokines were not significantly different between the two groups. The pulmonary cytokine levels in the 73-OR-infected group were higher than those in the 35-OR-infected group, which was consistent with the results of mouse plasma analysis (Figure 6C).

## Discussion

Previously, we had demonstrated that macrolide-resistant isolates mainly clustered in CC449 and CC363 although *M. catarrhalis* has a diverse genetic background [9]. *Mycobacterium tuberculosis* is reported to be associated with the fitness cost of antimicrobial resistance [8]. Hence, we hypothesize that the pathogenicity of macrolide-resistant isolates is higher than that of macrolide-susceptible isolates owing to efficient energy conservation. The role of macrolide susceptibility in the pathogenicity of *M. catarrhalis* to the host is unknown. This study compared the pathogenicity of 30 macrolide-resistant isolates and 40 macrolide-susceptible isolates. Based on previous studies, 46 essential virulence genes were extracted [15,27,28]. The distribution of these essential virulence genes in 70 *M. catarrhalis* isolates was analysed to examine its correlation with macrolide susceptibility. Additionally, the ability of bacterial clone isolates to adhere to A549 cells *in vitro* was comparatively analysed to establish its correlation with virulence genes. Based on *in vitro* studies, mice were infected with a pair of *M. catarrhalis* isolates with different pathogenic behaviours and macrolide susceptibilities. Pulmonary clearance, histological manifestations, and cytokine production were analysed in infected mice.

The prevalence of *uspA2*, *uspA2H*, *pilO*, *lbpB*, *lex1*, *modM*, *mboIA*, and *mboIB* significantly differed between the four clonal complexes and between the macrolide susceptibility groups. However, the roles of these genes in macrolide susceptibility or pathogenicity are unknown. To elucidate the potential correlation, this study aimed to examine these genes and their possible functions.

UspA2, an outer membrane protein, is involved in adherence to the extracellular matrix (ECM) [29] and serum resistance [30,31]. UspA2H, a hybrid of UspA1 and UspA2, is involved in adherence [12], biofilm formation [32], and serum resistance *in vitro* [30]. A previous study demonstrated that among respiratory tract infection isolates, the distribution of *uspA2* and *uspA2H* in children (95% and 5%, respectively) was significantly different from that in adults (61% and 39%, respectively) [33]. However, the distribution of *uspA2* and *uspA2H* was not markedly different between children (75.8% and 63.6%, respectively) and adults (62.2% and 56.8%, respectively) in this study. Additionally, *uspA2* and *uspA2H* were less detected in macrolide-susceptible groups or macrolide-susceptible clonal complexes (CC446 and CC224) than that in macrolide-resistant groups or macrolide-resistant clonal complexes (CC449 and CC363), indicating a negative correlation between *uspA2*/*uspA2H* prevalence and macrolide susceptibility. Based on the minimum inhibitory concentration values, 78.6% (22/28) and 67.9% (19/28) of *uspA2-*negative and *uspA2H-*negative isolates were susceptible to macrolide, respectively, while 42.9% (18/42) and 50.0% (21/42) of *uspA2-*positive and *uspA2H-*positive isolates were sensitive to macrolide (*P < *0.05), respectively.

PilO and PilN are integral inner membrane proteins that play an important role in type IV pilus formation, which is essential for pathogenicity, DNA transformation, and motility [34,35]. The distribution of *pilO* varied among the four clonal complexes (Table 1 and Figure 2). In particular, *pilO* was absent in CC446. However, *pilO* prevalence was not correlated with bacterial adhesion or invasion.

Lactoferrin binding protein B (LbpB), a bi-lobed outer membrane-bound lipoprotein, plays an essential role in the iron acquisition process in some gram-negative pathogens [36,37]. In this study, the prevalence of *lbpB* varied between the four clonal complexes (Table 1 and Figure 2) and between the macrolide susceptibility groups. The distribution of *lbpB*, which was specifically absent in CC224, significantly varied between the macrolide-resistant and macrolide-susceptible groups (*P* = 0.0006).

In *Haemophilus spp.*, Lex1 is involved in LOS biosynthesis, serum resistance, adherence, and invasion [38,39]. The function of the *lex1* in *M. catarrhalis*, has not been elucidated. In this study, the distribution of *lex1* varied between the four clonal complexes (Table 1 and Figure 2). In particular, *lex1* was absent in CC224, which mostly comprises macrolide-susceptible isolates (*P* < 0.05). However, *lex1* prevalence was not correlated with the ability of bacteria to adhere or invade A549 cells.

The modulation of the expression of ModM, a phase-variable type III DNA methyltransferase, affects the expression of various genes, known as a phase-variable regulon [40]. The three alleles of *modM* (*modM1, modM2,* and *modM3*) potentially regulate the expression of multiple genes associated with colonization, infection, and protection against host defences [41]. In this study, *modM* was detected in all isolates of CC446 and CC363 but not in those of CC224 and CC449. Additionally, *modM2* and *modM3* accounted for 96.8% (30/31) and 3.2% (1/31), respectively, of the detected *modM*. No *modM1* was detected in the isolates, which was consistent with the results of a previous study [41].

The genes *mboIA* and *mboIB* are always simultaneously present or completely absent. Previously, we had reported that *mboIA* and *mboIB* were associated with macrolide resistance [42], which was further confirmed in this study. However, the functions of *mboIA* and *mboIB* in *M. catarrhalis* are unknown. In this study, *mboIA* and *mboIB* genes were specifically absent in CC449. However, the correlation of the absence of *mboIA* and *mboIB* with the weak invasion ability of CC449 is unclear.

In this study, *M. catarrhalis* populations exhibited diverse abilities to adhere and invade A549 cells. Macrolide-resistant isolates exhibited enhanced adhesion (*P *= 0.0019) and suppressed invasion (*P < *0.0001) when compared with macrolide-susceptible isolates (Figure 3B and D). Similarly, the adhesion and invasion significantly varied among the clonal complexes. CC363, a predominant macrolide-resistant clonal complex, exhibited the strongest host cell adhesion ability. Meanwhile, CC446, a purely macrolide-susceptible clonal complex, exhibited the strongest host cell invasion ability (Figure 3C and E). Next, one macrolide-resistant isolate (35-OR) with strong host cell adhesion ability and a weak host cell invasive ability and a macrolide-susceptible isolate (73-OR) with weak host cell adhesion ability and a strong host cell invasive ability were selected to infect A549 cells. The differences in the host cell adhesion and invasion abilities of *M. catarrhalis* populations were examined using TEM (Figure 4). *M. catarrhalis* 35-OR aggregated on the A549 cell surface in a multicellular grape-like form (Figure 4A) without cellular invasion. In contrast, some *M. catarrhalis* 73-OR isolates with a diplococcus form adhered to the surface of A549 cells and infiltrated the cells (Figure 4B). These results were consistent with the bacterial growth analysis (adhesion and invasion assays) results.

Mouse experiments revealed differential changes in the lung tissues of the three groups. Compared with those in the control group, the number of inflammatory cells, especially infiltrated neutrophils and pulmonary macrophages, was higher and fibrous tissue hyperplasia was prominent in the infected groups. Meanwhile, inflammation severity in the 35-OR-infected group was higher than that in the 73-OR-infected group as evidenced by increased numbers of infiltrating inflammatory cells and fibrous tissue hyperplasia. The pulmonary clearance of the 73-OR isolate was rapid when compared with that of the 35-OR isolate (Figure 6). This was consistent with the histological (which revealed severe inflammation in the 35-OR-infected group) and TEM analyses. The WBC counts in the blood of the 73-OR-infected and 35-OR-infected groups were downregulated. In particular, the WBC counts were significantly downregulated in the 35-OR-infected group (*P *= 0.0393) (Figure 6B). We speculated that WBCs were rapidly recruited from the circulation to the injured lung tissues. Furthermore, the upregulated cytokines, such as Csf2, Il4, and Il13, which function in different phases of macrophage activation, in the lung tissues (Figure 7) and blood (Figure 6C), were associated with pulmonary macrophages [43]. The secretion of IL1B, IL6, TNF [44], and IL18 is triggered by activated macrophages, resulting in the recruitment of immune effector cells, such as neutrophils and monocytes and the death of pathogen-infected cells [45–47]. This explains the similar production levels of cytokines between the 35-OR-infected and 73-OR-infected groups in adhesion and invasion assays as no pulmonary macrophages were added. These findings indicate that pulmonary macrophages may play a critical role in the rapid immune response against *M. catarrhalis* infection. The immune response triggered by pulmonary macrophages may vary depending on *M. catarrhalis* populations. For example, the cytokine concentrations in the 73-OR-infected group were higher than those in the 35-OR-infected group. However, further studies are needed to examine the differential immune responses elicited by pulmonary macrophages against different *M. catarrhalis* populations.

*M. catarrhalis* populations exhibited diverse pathogenicity *in vitro* and *in vivo*. Adhesion and invasion are two consecutive steps in *M. catarrhalis* colonization and infection. Hence, the factors involved in the bacterial interaction with host cells, such as virulence genes, receptors, and cytokines must be determined.

One limitation of this study was that it focused only on four representative clonal complexes (CC449, CC363, CC224, and CC446) and determined their abilities to adhere and invade human epithelial cells and produce cytokines. Future studies must use isolates with diverse genetic backgrounds to verify the findings of this study. Additionally, the classical gene knockout and complementation experiments were not performed to examine the effect of virulence genes on bacterial ability to adhere and invade human respiratory epithelial cells. These experiments will be performed in the follow-up study.

## Supplementary Material

Supplemental Material

## Data Availability

The raw Illumina sequencing reads have been deposited in the Sequencing Read Archive database (https://www.ncbi.nlm.nih.gov/sra) (accession numbers: SRX2447596–SRX2447599, and SRX2161439, SRX2161445, SRX2161448, and SRX2161449).

## References

[1] Ventura F, Barranco R, Buffelli F, et al. Unexpected and sudden death Due to undiagnosed moraxella catarrhalis meningoencephalitis in a 40-day-old infant. Am J Forensic Med Pathol. 2020;41:333–337. 32618581 10.1097/PAF.0000000000000588

[2] Hirai J, Kinjo T, Koga T, et al. Clinical characteristics of community-acquired pneumonia due to *moraxella catarrhalis* in adults: a retrospective single-centre study. BMC Infect Dis. 2020;20:821. 33172398 10.1186/s12879-020-05564-9 PMC7653842

[3] National Health and Family Planning Commission Expert Committee on Rational Use of Medicines for Children Pharmaceutical Group. Investigation on the rational use of antibacterial agents by Chinese pediatricians in 2016. Zhonghua Er Ke Za Zhi. 2018;56:897–906. 30518003 10.3760/cma.j.issn.0578-1310.2018.12.004

[4] Liu Y, Zhao C, Zhang F, et al. High prevalence and molecular analysis of macrolide-nonsusceptiblemoraxella catarrhalisisolated from nasopharynx of healthy children in China. Microb Drug Resist. 2012;18:417–426. 22394083 10.1089/mdr.2011.0175

[5] Liu Y, Xu H, Xu Z, et al. High-Level macrolide-resistant *moraxella catarrhalis* and development of an allele-specific PCR assay for detection of 23S rRNA gene A2330T mutation: A three-year study at a Chinese tertiary hospital. Microb Drug Resist. 2015;21:507–511. 25923017 10.1089/mdr.2014.0217

[6] Richard MP, Aguado AG, Mattina R, et al. Sensitivity to sparfloxacin and other antibiotics, of *streptococcus pneumoniae*, *haemophilus influenzae* and *moraxella catarrhalis* strains isolated from adult patients with community-acquired lower respiratory tract infections: a European multicentre study. SPAR study group. surveillance programme of antibiotic resistance. J Antimicrob Chemother. 1998;41:207–214. 9533462 10.1093/jac/41.2.207

[7] Andersson DI, Hughes D. Antibiotic resistance and its cost: is it possible to reverse resistance? and its cost: is it possible to reverse resistance? Nat Rev Microbiol. 2010;8(4):260–271. 20208551 10.1038/nrmicro2319

[8] Cohen T, Sommers B, Murray M. The effect of drug resistance on the fitness of *mycobacterium tuberculosis*. Lancet Infect Dis. 2003;3:13–21. 12505028 10.1016/s1473-3099(03)00483-3

[9] Liu YL, Xiao M, Cheng JW, et al. Macrolide-Resistant isolates Are highly concentrated in Two MLST clonal complexes -CCN10 and CC363. Front Microbiol. 2017;8:201. 28239374 10.3389/fmicb.2017.00201 PMC5300973

[10] Du Y, Zhou H, Wang F, et al. Multilocus sequence typing-based analysis of *moraxella catarrhalis* population structure reveals clonal spreading of drug-resistant strains isolated from childhood pneumonia. Infect Genet Evol. 2017;56:117–124. 29155241 10.1016/j.meegid.2017.11.018

[11] Holm MM, Vanlerberg SL, Sledjeski DD, et al. The Hag protein of *moraxella catarrhalis*strain O35E Is associated with adherence to human lung and middle Ear cells. Infect Immun. 2003;71:4977–4984. 12933840 10.1128/IAI.71.9.4977-4984.2003 PMC187358

[12] Lafontaine ER, Cope LD, Aebi C, et al. The UspA1 protein and a second type of UspA2 protein mediate adherence of *moraxella catarrhalis* to human epithelial cells In vitro. J Bacteriol. 2000;182:1364–1373. 10671460 10.1128/jb.182.5.1364-1373.2000 PMC94425

[13] Pearson MM, Lafontaine ER, Wagner NJ, et al. A *hag* mutant of *moraxella catarrhalis* strain O35E Is deficient in hemagglutination, autoagglutination, and immunoglobulin D-binding activities. Infect Immun. 2002;70:4523–4533. 12117964 10.1128/IAI.70.8.4523-4533.2002 PMC128162

[14] Timpe JM, Holm MM, Vanlerberg SL, et al. Identification of a*moraxella catarrhalis*outer membrane protein exhibiting both adhesin and lipolytic activities. Infect Immun. 2003;71:4341–4350. 12874311 10.1128/IAI.71.8.4341-4350.2003 PMC166007

[15] Su Y-C, Singh B, Riesbeck K. Moraxella catarrhalis: from interactions with the host immune system to vaccine development. Future Microbiol. 2012;7:1073–1100. 22953708 10.2217/fmb.12.80

[16] Bankevich A, Nurk S, Antipov D, et al. SPAdes: a new genome assembly algorithm and its applications to single-cell sequencing. J Comput Biol. 2012;19:455–477. 22506599 10.1089/cmb.2012.0021 PMC3342519

[17] Seemann T. Prokka: rapid prokaryotic genome annotation. Bioinformatics. 2014;30:2068–2069. 24642063 10.1093/bioinformatics/btu153

[18] Inouye M, Dashnow H, Raven L-A, et al. SRST2: rapid genomic surveillance for public health and hospital microbiology labs. Genome Med. 2014;6:90. 25422674 10.1186/s13073-014-0090-6 PMC4237778

[19] Page AJ, Cummins CA, Hunt M, et al. Roary: rapid large-scale prokaryote pan genome analysis. Bioinformatics. 2015;31:3691–3693. 26198102 10.1093/bioinformatics/btv421 PMC4817141

[20] Ye J, Zhang Y, Cui H, et al. WEGO 2.0: a web tool for analyzing and plotting GO annotations, 2018 update. Nucleic Acids Res. 2018;46:W71–W75. 29788377 10.1093/nar/gky400 PMC6030983

[21] Zhao Y, Wang J, Chen J, et al. A literature review of gene function prediction by modeling gene ontology. Front Genet. 2020;11:400. 32391061 10.3389/fgene.2020.00400 PMC7193026

[22] Slevogt H, Seybold J, Tiwari KN, et al. *Moraxella catarrhalis* is internalized in respiratory epithelial cells by a trigger-like mechanism and initiates a TLR2- and partly NOD1-dependent inflammatory immune response. Cell Microbiol. 2007;9:694–707. 17054439 10.1111/j.1462-5822.2006.00821.x

[23] Inoue S, Nakamura H, Otake K, et al. Impaired pulmonary inflammatory responses are a prominent feature of *streptococcal pneumonia* in mice with experimental emphysema. Am J Respir Crit Care Med. 2003;167:764–770. 12598218 10.1164/rccm.2105111

[24] Jones MM, Johnson A, Koszelak-Rosenblum M, et al. Role of the oligopeptide permease ABC transporter of *moraxella catarrhalis* in nutrient acquisition and persistence in the respiratory tract. Infect Immun. 2014;82:4758–4766. 25156736 10.1128/IAI.02185-14 PMC4249328

[25] Murphy TF, Brauer AL, Johnson A, et al. ATP-Binding Cassette (ABC) transporters of the human respiratory tract pathogen, *moraxella catarrhalis*: role in virulence. PloS one. 2016;11:e0158689. 27391026 10.1371/journal.pone.0158689 PMC4938438

[26] Singh B, Alvarado-Kristensson M, Johansson M, et al. The respiratory pathogen *moraxella catarrhalis* targets collagen for maximal adherence to host tissues. mBio. 2016;7:e00066. 27006460 10.1128/mBio.00066-16 PMC4807357

[27] Blakeway LV, Tan A, Peak IRA, et al. Virulence determinants of *moraxella catarrhalis*: distribution and considerations for vaccine development. Microbiology. 2017;163:1371–1384. 28893369 10.1099/mic.0.000523

[28] Riesbeck K. Complement evasion by the human respiratory tract pathogens *haemophilus influenzae* and *moraxella catarrhalis*. FEBS Lett 2020;594:2586–2597. 32053211 10.1002/1873-3468.13758

[29] Tan TT, Nordström T, Forsgren A, et al. The respiratory pathogen *moraxella catarrhalis* adheres to epithelial cells by interacting with fibronectin through ubiquitous surface proteins A1 and A2. J Infect Dis. 2005;192:1029–1038. 16107956 10.1086/432759

[30] Singh B, Al-Jubair T, Voraganti C, et al. *Moraxella catarrhalis* binds plasminogen To evade host innate immunity. Infect Immun. 2015;83:3458–3469. 26099590 10.1128/IAI.00310-15 PMC4534650

[31] Liu G, Ermert D, Johansson ME, et al. PRELP enhances host innate immunity against the respiratory tract pathogen *moraxella catarrhalis*. J Immunol. 2017;198:2330–2340. 28148731 10.4049/jimmunol.1601319

[32] Pearson MM, Hansen EJ. Identification of gene products involved in biofilm production by *moraxella catarrhalis* ETSU-9 In vitro. Infect Immun. 2007;75:4316–4325. 17562762 10.1128/IAI.01347-06 PMC1951151

[33] Verhaegh SJC, Streefland A, Dewnarain JK, et al. Age-related genotypic and phenotypic differences in *moraxella catarrhalis* isolates from children and adults presenting with respiratory disease in 2001-2002. Microbiology. 2008;154:1178–1184. 18375810 10.1099/mic.0.2007/015057-0

[34] Karuppiah V, Collins RF, Thistlethwaite A, et al. Structure and assembly of an inner membrane platform for initiation of type IV pilus biogenesis. Proc Natl Acad Sci U S A. 2013;110:E4638–E4647. 24218553 10.1073/pnas.1312313110 PMC3845092

[35] Leighton TL, Mok MC, Junop MS, et al. Conserved, unstructured regions in *pseudomonas aeruginosa* PilO are important for type IVa pilus function. Sci Rep. 2018;8:2600. 29422606 10.1038/s41598-018-20925-w PMC5805733

[36] Bonnah RA, Wong H, Loosmore SM, et al. Characterization of*moraxella*(*branhamella*)*catarrhalis lbpB*,*lbpA*, and lactoferrin receptor *orf3* isogenic mutants. Infect. Infect Immun. 1999;67:1517–1520. 10024604 10.1128/iai.67.3.1517-1520.1999 PMC96490

[37] Ostan NKH, Yu RH, Ng D, et al. Lactoferrin binding protein B - a bi-functional bacterial receptor protein. PLoS Pathog. 2017;13:e1006244. 28257520 10.1371/journal.ppat.1006244 PMC5352143

[38] Ma D, Alberti M, Lynch C, et al. The local repressor AcrR plays a modulating role in the regulation of acrAB genes of *escherichia coli* by global stress signals. Mol Microbiol. 1996;19:101–112. 8821940 10.1046/j.1365-2958.1996.357881.x

[39] Zhou Q, Feng S, Zhang J, et al. Two glycosyltransferase genes of SC096 implicated in lipooligosaccharide biosynthesis, serum resistance, adherence, and invasion. Front Cell Infect Microbiol. 2016;6:100. 27672622 10.3389/fcimb.2016.00100 PMC5018477

[40] Seib KL, Peak IRA, Jennings MP. Phase variable restriction-modification systems in *moraxella catarrhalis*. FEMS Immunol Med Microbiol. 2002;32:159–165. 11821238 10.1111/j.1574-695X.2002.tb00548.x

[41] Blakeway LV, Power PM, Jen FEC, et al. Modm DNA methyltransferase methylome analysis reveals a potential role for *moraxella catarrhalis* phasevarions in otitis media. FASEB J. 2014;28:5197–5207. 25183669 10.1096/fj.14-256578

[42] Liu YL, Li DF, Xu HP, et al. Use of next generation sequence to investigate potential novel macrolide resistance mechanisms in a population of *moraxella catarrhalis* isolates. Sci Rep. 2016;6:35711. 27774989 10.1038/srep35711 PMC5075928

[43] Gordon S, Martinez FO. Alternative activation of macrophages: mechanism and functions. Immunity. 2010;32:593–604. 20510870 10.1016/j.immuni.2010.05.007

[44] Laurent S, Monique S, Viviane B, et al. Lack of IL-10 synthesis by murine alveolar macrophages upon lipopolysaccharide exposure. comparison with peritoneal macrophages. J Leukoc Biol. 2000;67:545–552. 10770288 10.1002/jlb.67.4.545

[45] Thomas PG, Dash P, Aldridge JR, et al. The intracellular sensor NLRP3 mediates key innate and healing responses to influenza A virus via the regulation of caspase-1. Immunity. 2009;30:566–575. 19362023 10.1016/j.immuni.2009.02.006 PMC2765464

[46] Tisoncik JR, Korth MJ, Simmons CP, et al. Into the eye of the cytokine storm. Microbiol Mol Biol Rev. 2012;76:16–32. 22390970 10.1128/MMBR.05015-11 PMC3294426

[47] Wang CH, Liu CY, Wan YL, et al. Persistence of lung inflammation and lung cytokines with high-resolution CT abnormalities during recovery from SARS. Respir Res. 2005;6:42. 15888207 10.1186/1465-9921-6-42 PMC1156954

